# Vacunas contra el SARS-CoV-2: ¿son una realidad para América Latina?

**DOI:** 10.7705/biomedica.5826

**Published:** 2020-06-30

**Authors:** Alfredo G. Torres

**Affiliations:** 1 Department of Microbiology and Immunology, Sealy Center for Vaccine Development, University of Texas Medical Branch, Galveston, TX, USA University of Texas Medical Branch GalvestonTX USA

*Ha vuelto una pandemia. A pesar de los grandes avances de la ciencia y el tan celebrado ingenio de nuestra especie, nuestra mejor defensa hasta ahora es simplemente quedarnos en casa, escondidos en nuestras cuevas para que el depredador no nos encuentre. Para los que al menos tengan un poco de humildad, es un momento de reflexión. Para los demás, es solo una cosa más que aniquilar*. 

Rodrigo García, “Carta a mi padre, Gabriel García Márquez”, New York Times, 6 de mayo del 2020

Desde la aparición del coronavirus SARS-CoV-2 en China en diciembre del 2019 y el anuncio de la Organización Mundial de la Salud de que la enfermedad causada por el virus se conocería como COVID-19, en febrero del 2020, la pandemia ha seguido avanzando a nivel mundial. En septiembre del 2020 se reportó que los casos de infección por el SARS-CoV-2 habían superado los 28 millones y las 917.000 muertes. 

La crisis sanitaria generada por el SARS-CoV-2 y el impacto que está teniendo en la economía mundial ejercen presión sobre las naciones afectadas, así que ha movilizado a los diferentes grupos de investigación y a la industria farmacéutica para acelerar los esfuerzos y encontrar una cura para la COVID-19. Aunque algunos avances se han obtenido en un corto periodo en la identificación de agentes terapéuticos que ayudan a combatir la infección, ningún medicamento ni producto biológico ha demostrado hasta el momento una efectividad total para eliminar el coronavirus.

Por ello, los grupos de investigación de todo el mundo han enfocado sus esfuerzos en la búsqueda de una vacuna que permita contener la pandemia. La colaboración entre el sector público, las universidades y el sector privado se ha convertido en una actividad esencial en la carrera para hallarla. Los expertos hablan de que se necesitarán entre 12 y 18 meses para contar con una primera vacuna que haya sido evaluada desde la fase preclínica hasta la etapa clínica (fase 3), lo que permitiría emitir una aprobación de urgencia para empezar a producirla masivamente. Este avance acelerado se ha logrado gracias a que los investigadores están basando el diseño de las vacunas experimentales contra la COVID-19 en conocimientos obtenidos al desarrollarlas contra otros coronavirus. Dicho aprendizaje ha permitido la identificación rápida de epítopos específicos para el blanco, los cuales permitirían producir anticuerpos neutralizadores. Además, las plataformas que se están utilizando logran inducir tanto la reacción efectiva de las célulasT como la de potentes anticuerpos neutralizadores y pueden incorporar, asimismo, productos adyuvantes que mejoran la inmunogenicidad.

La carrera para producir una vacuna efectiva contra la COVID-19 está entrando en las etapas finales de los estudios clínicos en humanos: hasta el 29 de julio pasado, el panorama de las vacunas candidatas incluía 25 en fase de evaluación clínica y 139 en fase de evaluación preclínica. Al menos cuatro de ellas son las que están más cerca de probar su efectividad y seguridad en la fase clínica 3, por lo que es importante que el lector conozca con mayor detalle cómo están hechas y qué probabilidad hay de recibir alguna de ellas. Las vacunas experimentales más avanzadas en su evaluación clínica son las producidas por Sinovac Biotech (China), la conocida como ChAdOx1 nCoV- 19 de la Universidad de Oxford/AstraZeneca (Reino Unido), la desarrollada por la compañía Moderna (Estados Unidos) y la de la compañía Pfizer en colaboración con BioNTech (Estados Unidos).

La vacuna de Sinovac Biotech está siendo probada en Brasil en colaboración con el Instituto Butantan, el principal centro inmunológico de referencia de ese país, cuyo objetivo es llegar a probar la vacuna en 9.000 voluntarios. La “CoronaVac” se basa en partículas inactivadas del virus SARS-CoV-2 que no producen la enfermedad, pero sí permiten generar una reacción inmunitaria óptima. Si la vacuna resulta segura y efectiva en los estudios clínicos de fase 3, el Instituto Butantan tendrá el derecho de producir 120 millones de dosis, de las cuales Brasil podrá contar con 60 millones para su distribución sin tener que comprarla en el exterior. Otros países de Latinoamérica, como Chile, han firmado convenios con esta compañía para probar la vacuna en su población y, así, tener la opción de contar, por lo menos, con 20 millones de dosis el próximo año, una vez se obtenga la licencia.

La vacuna de Oxford/AstraZeneca, también conocida como AZD1222, está diseñada a partir de un virus genéticamente modificado que causa resfriado común en chimpancés pero que no causa infecciones en los humanos, aunque la modificación genética lo haya hecho más semejante al coronavirus. Algunos resultados publicados recientemente demostraron que esta vacuna tiene la capacidad de generar anticuerpos neutralizadores y células T con una sola dosis, según las pruebas hechas en cerca de 1.000 voluntarios. Actualmente, se están enrolando 8.000 voluntarios para participar en la fase 3 que se llevará a cabo en el Reino Unido. La compañía AstraZeneca tiene la licencia para producir la vacuna, lo que incluye un acuerdo para producir miles de millones de dosis con el Instituto de Sueros de la India, con la intención de distribuirla en el 2021 en países de bajos y medianos recursos a un costo estimado de € 2,5 la unidad. Entre los gobiernos latinoamericanos que han firmado acuerdos con esta compañía, está el brasileño, que acordó adquirir 100 millones de dosis una vez se tenga la licencia para su distribución.

Las vacunas de Moderna y Pfizer se basan en una nueva tecnología que permite su desarrollo y manufactura más rápidamente que los métodos tradicionales, pero este tipo de vacuna no se ha aprobado antes para su uso en humanos. La tecnología utiliza ARN mensajero, o lo que se conoce como ARN mensajero sintético (mRNA), que no causa infección ni síntomas asociados con la COVID19, pero que, al inyectarse e introducirse en las células humanas, genera un fragmento de la proteína de la espícula del coronavirus, lo que es suficiente para que se dé la reacción inmunitaria.

La vacuna de Moderna se conoce como mRNA-1273. En un estudio clínico dirigido por el *National Institute of Allergy* and *Infectious Diseases* (NIAD) de Estados Unidos, la vacuna resultó en general segura y fue bien tolerada por los participantes, que desarrollaron anticuerpos neutralizadores contra el virus. Esta vacuna es parte del proyecto del gobierno de este país conocido como *Operation Warp Speed* (operación máxima velocidad), diseñado para acelerar la producción de una vacuna y tener 300 millones de dosis seguras y efectivas para enero de 2021. Esta vacuna se encuentra en etapa de evaluación clínica de fase 3, en 30.000 adultos y 89 sitios de investigación clínica. El acceso a ella en Latinoamérica será limitado hasta que los Estados Unidos hayan completado el número de dosis necesarias para su población.

La vacuna de Pfizer, conocida como BNT162b2, ha recibido la aprobación *fast track* de la *Federal Drug Administration* de los Estados Unidos (FDA), lo que permite acelerar la producción y la investigación, y puede repercutir en la rapidez con la que saldrá al mercado. Los estudios clínicos iniciales con distintas dosis de la vacuna demostraron que los títulos de anticuerpos que se produjeron fueron mayores que los de los pacientes que se han recuperado de la COVID-19. Pfizer inició los estudios clínicos de la fase 3 en julio de este año con 30.000 voluntarios y, si los resultados son positivos, se ha acordado la producción de cerca de 100 millones de dosis para finales del 2020 y de 1.300 millones de dosis para finales del próximo año. Además de los Estados Unidos, el único país en Latinoamérica que estableció un acuerdo con BioNTech-Pfizer es Argentina, donde se llevará a cabo una de las fases clínicas para probar la efectividad de la vacuna.

Una vez se demuestre que una de estas vacunas es segura y efectiva, la única barrera que quedaría por vencer para producir suficientes dosis sería la capacidad mundial para hacerlo. Infortunadamente, hay barreras artificiales en el camino de la vacuna. Las leyes de propiedad intelectual confieren a las compañías farmacéuticas los derechos exclusivos para producir las vacunas por cierto número de años con el objetivo de ayudar a que recuperen el costo de la inversión y la innovación. En muchas ocasiones, sin embargo, se abusa de los derechos de propiedad intelectual y se crean monopolios, que, en el caso de la vacuna contra la COVID-19, resultaría en una amenaza real que limitaría su acceso y causaría desabastecimiento y retrasos innecesarios en su producción, lo que sería letal para la población.

Los países latinoamericanos deben acelerar las conversaciones con la Organización Mundial de la Salud, las compañías farmacéuticas y otras organizaciones internacionales para garantizar el acceso inmediato a una o varias de las vacunas disponibles u obtener los derechos para iniciar la producción interna de suficientes dosis que les permita empezar a proteger a su población más propensa.

